# The international clinical trials registry platform (ICTRP): data integrity and the trends in clinical trials, diseases, and drugs

**DOI:** 10.3389/fphar.2023.1228148

**Published:** 2023-09-18

**Authors:** Eugenia D. Namiot, Diana Smirnovová, Aleksandr V. Sokolov, Vladimir N. Chubarev, Vadim V. Tarasov, Helgi B. Schiöth

**Affiliations:** ^1^ Department of Surgical Sciences, Division of Functional Pharmacology and Neuroscience, Uppsala University, Uppsala, Sweden; ^2^ Advanced Molecular Technology, Limited Liable Company (LLC), Moscow, Russia

**Keywords:** ICTRP, DrugBank, hidden duplicates, clinical trials, trends ICTRP, trends

## Abstract

**Introduction:** Clinical trials are the gold standard for testing new therapies. Databases like ClinicalTrials.gov provide access to trial information, mainly covering the US and Europe. In 2006, WHO introduced the global ICTRP, aggregating data from ClinicalTrials.gov and 17 other national registers, making it the largest clinical trial platform by June 2019. This study conducts a comprehensive global analysis of the ICTRP database and provides framework for large-scale data analysis, data preparation, curation, and filtering.

**Materials and methods:** The trends in 689,793 records from the ICTRP database (covering trials registered from 1990 to 2020) were analyzed. Records were adjusted for duplicates and mapping of agents to drug classes was performed. Several databases, including DrugBank, MESH, and the NIH Drug Information Portal were used to investigate trends in agent classes.

**Results:** Our novel approach unveiled that 0.5% of the trials we identified were hidden duplicates, primarily originating from the EUCTR database, which accounted for 82.9% of these duplicates. However, the overall number of hidden duplicates within the ICTRP seems to be decreasing. In total, 689 793 trials (478 345 interventional) were registered in the ICTRP between 1990 and 2020, surpassing the count of trials in ClinicalTrials.gov (362 500 trials by the end of 2020). We identified 4 865 unique agents in trials with DrugBank, whereas 2 633 agents were identified with NIH Drug Information Portal data. After the ClinicalTrials.gov, EUCTR had the most trials in the ICTRP, followed by CTRI, IRCT, CHiCTR, and ISRCTN. CHiCTR displayed a significant surge in trial registration around 2015, while CTRI experienced rapid growth starting in 2016.

**Conclusion:** This study highlights both the strengths and weaknesses of using the ICTRP as a data source for analyzing trends in clinical trials, and emphasizes the value of utilizing multiple registries for a comprehensive analysis.

## 1 Introduction

Clinical trials remain the golden standard for evaluating the efficacy of newly proposed therapeutic agents. Pharmacological development has prompted increase in novel drugs that require assessment, along with the exploration of new indications, leading to a significant rise in the number of clinical trials. Several databases containing registries of clinical trials have been set up to organize information and to ease access to the general public and healthcare/government authorities. In 2,000, as a part of the first U.S. Federal law regulating trial registries and making them obligatory, the ClinicalTrials.gov database was created by the NIH US library of Medicine (Merrill, 1999; Zarin et al., 2011; DeVito et al., 2020). Despite being one of the most used databases, ClinicalTrials.gov has some limitations, one of them being that it primarily contains information on U.S. and European trials. Therefore in 2006, the World Health Organization (WHO) set up the International Clinical Trials Registry Platform (ICTRP) to accumulate clinical trial data from multiple clinical trial registries to generate a view of the clinical trials worldwide with an accessible search portal (World Health Organization, 2018; Negoro et al., 2019). This globally-oriented platform provides information on trials listed in ClinicalTrials.gov, as well as trials from seventeen other registries worldwide, referred to here as non-ClinicalTrials.gov trials. As of June 2019, ICTRP is recognized as the largest clinical trial platform (Juneja et al., 2019).

Analyzing such a significant amount of clinical trial data requires advanced methods. Some data extraction steps can be accomplished relatively easily with the assistance of machine learning methods or regular programming languages and tools (Vamathevan et al., 2019; Dronkers, 2020; Edem et al., 2021). However, the problem of removing duplicates has not yet been completely solved. One important problem is that trials can be registered multiple times and these are known as “duplicates” and are usually flagged by the ICTRP platform. Trials that were not identified by the system can be referred to as “hidden duplicates” (van Valkenhoef et al., 2016; Kumari et al., 2020). In a 2016 study, the authors noted that nearly 45% of all duplicates in the ICTRP were not identified by the system, seriously affecting the analysis of clinical trials (out of a sample of 434 pairs) (van Valkenhoef et al., 2016). The problem of hidden duplicates can impact the perception of clinical trial trends in a particular area or globally, hindering the identification of the actual number of trials. To date, the World Health Organization (WHO) has published guidelines for identifying and managing duplicates in clinical trial registries, which could help address this issue. However, the available literature lacks detailed information on the precise methods used to identify hidden duplicates. One possible approach was presented by van Valkenhoef and others in 2016, which involves two crucial steps: developing a scoring model based on text-similarity methods, and manually reviewing the registries with high scores (van Valkenhoef et al., 2016). In a recent review from 2020, a model was developed to identify true pairs by comparing common entries across all clinical trial databases, such as scientific and public titles, phases, conditions, and outcome measures, using a string-match method (Kumari et al., 2020). In 2021, another group of researchers used several methods, including a random forest classifier and decision trees, to improve the deduplication process and increase the precision and accuracy of predictions compared to regular study ID matching (Thiele et al., 2021). Despite these efforts, several authors emphasize the need for further development of methods and research into the deduplication process (van Valkenhoef et al., 2016; Kumari et al., 2020; Saberwal, 2021).

There are obviously number of other integrity issues that are important to consider when analyzing such large data. Important issues relate to the completeness and relevance of the ICTRP data when compared with other registries and databases. Several studies have pointed out geographical and other differences in clinical trials within the same region (Ginn et al., 2018; Banno et al., 2019; Deinsberger et al., 2020). For instance, a study in 2020 revealed that the United States had the highest percentage of registered observational studies with pluripotent stem cells (41.6%), while China and Germany had much lower percentage (5%). However, it is important to note that China had fewer trials registered overall in this area. Similarly, gene therapy had a large number of clinical trials registered in the United States (63.3%, 1,643), with other countries having much lower percentage, for example, Australia and Spain having 1.2% (Ginn et al., 2018). The ICTRP database is therefore able to amalgamate databases from different countries mitigating the geographical bias. The number of analyses conducted on clinical trials is increasing. These analyses provide valuable insights into trends and the consequences of regulatory differences, such as the impact of orphan drugs on pharmaceutical development (Hauser et al., 2017). Many of the analysis of clinical trials are often based on the clinicaltrials.gov database and focus primarily on specific areas of medicine (e.g., antidiabetic therapy or Alzheimer’s disease drugs) (Bachurin et al., 2017; Anwar et al., 2019; Huang et al., 2020; Ji et al., 2020; Sokolov et al., 2021; Bondarev et al., 2022; Dahlén et al., 2022; Dambrova et al., 2022; Jovic et al., 2022; Kim et al., 2022; Namiot et al., 2022; Nazarova et al., 2022; Niemi et al., 2022; Namiot et al., 2023). However, some of these analyses have recognized the emergence of ICTRP, Clinical Trials Registry India (CTRI), and The European Union Clinical Trials Registry (EUCTR) as the primary source for trial searches, particularly in relationship to the global COVID-19 pandemic, which required analyzing global trends (Ji et al., 2020; Rao et al., 2021; Kim et al., 2022). While focusing on specific diseases can provide a deeper understanding of the issue, a lack of research on general trends has been noted. Only one study, which analyzed ICTRP trials registered up to 31 December 2013, focused on global trends showing a more gradual increase in trial registration in Asia due to certain regulation issues with a special focus on India and Japan (Viergever and Li, 2015).

In this study, we present a comprehensive global analysis of the ICTRP database, along with a methodology to facilitate large-scale analysis of the data through data preparation, curation, and filtering. Furthermore, we propose a straightforward algorithm to eliminate hidden duplicates from the clinical trial data. Additionally, we examine the trends in ICTRP and its sub-registries and compare them with the widely-used and referenced platform, ClinicalTrials.gov.

## 2 Materials and methods

### 2.1 Data collection

The complete ICTRP dataset was retrieved in the CSV format with the time stamp of 24th of May 2021 from the ICTRP platform. Clinical trials with a registration date between 1990 and 2020 were extracted. The dataset included 20 different types of information. The following columns have been used in this project: trial ID, secondary IDs, public title, scientific title, study type, study design, phase, registration date, enrollment date, target size, recruitment status, primary sponsor, countries, conditions, interventions and “bridged type”.

The full ICTRP dataset was loaded into a relational database to store the data. PostgreSQL 9.6 version was used as the relational database. Besides the ICTRP dataset, MESH dataset, NIH drug datasets, and Drug Bank datasets were imported into the relational database.

Medical Special Headings (MESH) database is an online platform that contains all common categories and their entry terms. It was used to set up a system to categorize the clinical trials by conditions. The Medical Special Headings version 2021 was used in this project (Lipscomb, 2000). Initially, it contained the columns Category and EntryTerms, which are synonym terms for each disease or condition. MESH Categories table was merged with the conditions section of the ICTRP table, creating the Conditions table that contained matched conditions for each trial ID. Only MESH categories in the following groups have been kept in the dataset and used in the project: Diseases [C], Behavior and Behavior Mechanisms [F01], and Mental Disorders [F03]. Other non-relevant to disease categories were excluded.

Drug names, categories, and synonyms coming from the Drug Information Portal of the National Library of Medicine (NIH DIP) were used to identify and categorize drugs used in clinical trials (Hochstein et al., 2009). The NIH Drug Information Portal was an online platform containing information about drugs, alternative names, categories, etc. We web-scraped the list of drugs, their synonyms and categories, using custom R-based scripts. The synonyms were matched with the interventions section of the ICTRP dataset, creating the Interventions table. The matched interventions were used in the process of identifying hidden duplicates. 140 DrugBank datasets were used to identify approved and investigational drugs in line with our previous studies [36—38]. DrugBank is a comprehensive platform consisting of information on drugs, their targets, enzymes and dosage forms among other data (Wishart et al., 2018). The DrugBank datasets were read in with the R package dbparser, importing datasets containing the following information: drugbank_id, type, name, and synonym (Ali and Ezzat, 2020). These datasets were then imported into the relational database enabling easy merging.

### 2.2 Data pre-processing

To perform a systematic analysis of clinical trials, several pre-processing steps were used to prepare the data. First, clinical trials without a registration date were excluded from further processing. The ICTRP dataset also contained visible and hidden duplicates. Duplicates in the ICTRP platform could occur when one individual clinical trial was registered onto the platform via multiple registries. Visible duplicates are duplicates that are flagged as duplicates by the ICTRP platform itself. To exclude these kinds of duplicates, records with “Child” in the data item “Bridged_type” were identified and eliminated from further processing.

**FIGURE 1:**
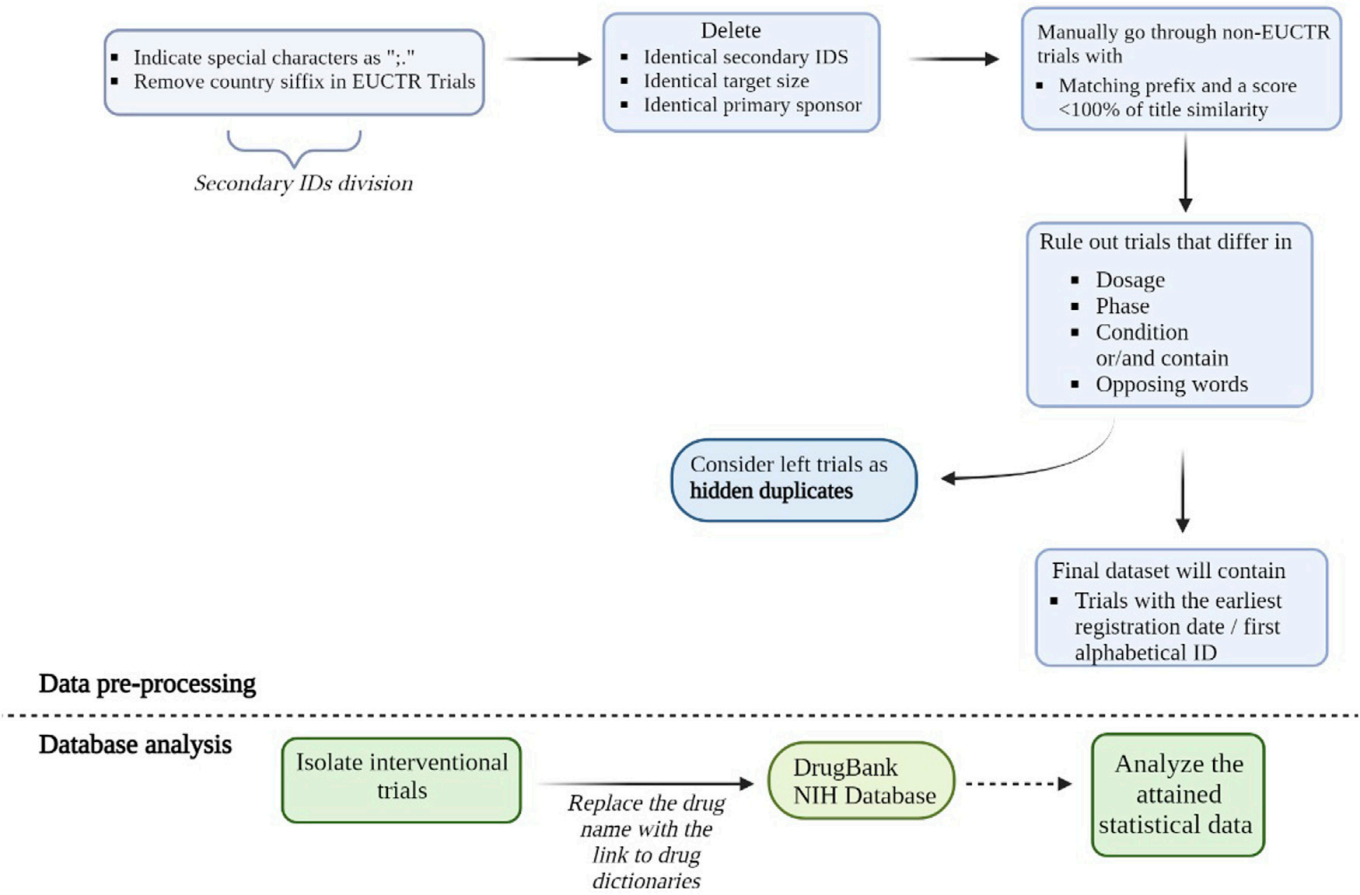
Pre-processing steps of the deduplication process. Hidden duplicates are duplicates not marked by the ICTRP platform as duplicates. To identify them we matched trials with an identical secondary ID, target size, primary sponsor, NIH drug name, or a matched compressed and lowercased intervention of the interventions column (for non-matched records). We also grouped trials that had at least 80% % similarity of concatenated public and scientific titles which was calculated using Levenshtein distance. Trials that did not contain the same EUCTR prefix and did not have similar titles were checked manually. Among this dataset, we then searched for trials with different dosages, phases and conditions and considered them as non-duplicates. All the left trials 833 were considered to be hidden duplicates. The final dataset for further analysis will contain trials with the earliest registration date and/or first alphabetical ID. Below the dashed line, we briefly summarized the databases analysis process. Using DrugBank and NIH databases we identified the exact agents and drug categories used in clinical trials. We then collected statistical data on identified agents, including unique agents for further analysis of general trends in either clinicaltrials.gov or non-clinicaltrials.gov databases. Such analysis also allows us to compare the efficacy of NIH and DrugBank drug databases by comparing the number of identified drugs using each of them.

The next step was to identify the hidden duplicates (Figure 1). Hidden duplicates are duplicates that are not flagged by the ICTRP platform as a duplicate and so they remain unnoticed and require special curation. Previous research on hidden duplicates in the ICTRP platform showed that the real number of duplicates could be twice as high as the number of duplicates that are currently flagged by the platform. To identify these hidden duplicates, an adapted modified method from Van Valkenhoef et al. (2016) was used (van Valkenhoef et al., 2016). First, clinical trials were matched by identical secondary IDs, using the secondary ID field. The secondary ID field was separated by the characters ";,." and secondary IDs containing the trial ID from the European Clinical Trials Register (EUCTR) were stripped of the country suffix to allow correct matching. The trials with a matching secondary ID were identified and the grouped records were investigated further. Besides matching secondary ID, trials were selected that had a matching target size, primary sponsor, at least 80% similarity of concatenated public and scientific title, a matched NIH drug name, or a matched compressed and lowercased intervention of the interventions column (for non-matched records). The similarity score of the concatenated titles was calculated using the Levenshtein distance algorithm. The algorithm is based on the difference between two string sequences, by calculating the minimum number of edits needed to change the first string into the second one (Navarro, 2001).

The hidden duplicate identification process also involved manually checking the trials, with title similarity of below 100% that did not contain the same EUCTR prefix. The title and condition columns were scanned and trials containing opposing words in these columns, such as “fasting” and “fed” or different dosage, phase or condition mentioned, were considered non-duplicates. The final selection of trials was considered as true hidden duplicates.

Certain variables were grouped before they have been used in the project. Disease conditions falling under multiple categories were assigned to a single primary category. Matched drugs could have been categorized into one or more drug groups as follows: approved, illicit, withdrawn, investigational, experimental, nutraceutical, or vet-approved. Vet-approved categories have not been taken into account and excluded from the analysis. If a drug had been approved anywhere, it was considered to be “approved”. If a drug had been withdrawn at some point, it was categorized as “withdrawn”. Investigational and experimental drugs have been categorized into one category. Drugs were also categorized by their drug categories using the categories from the NIH Drug Information Portal.

Furthermore, if a trial consisted of two mentioned phases, the phase was categorized into the earliest phase mentioned. Target size was categorized as follows: “0–10”, “11–50”, “51–100”, “101–1,000”or “>1,000”. The recruitment status was categorized as ―completed‖ when the word “complete” was mentioned in the recruitment status column. For recruitment status of “recruiting”, “open public recruiting”, “open to recruitment”, “enrolling by invitation”, “authorised-recruitment may be ongoing or finished”, “ongoing”, “approved for marketing”, “not yet recruiting” or “active, not recruiting”, it was categorized as “Active”. The Trial was categorized as “Not Active” if recruitment status contained “terminate”, “withdrawn”, “stopped”, “suspended”, “pending”, “not recruiting”, and “closed to recruitment of participant”. Trials with a missing or not done recruitment status were categorized as unknown. Allocation was either “Randomized” when some form of randomization was mentioned, “Nonrandomized” for nonrandomized trials and category “Unknown” if it was not specified. Masking was either “Blinded”, “Open Label” or “Unknown”. If a drug treatment was matched with the DrugBank synonyms, it was categorized as “Yes” and others were categorized as “No”.

### 2.3 Database analysis

We used the ICTRP_preprocessed_trials_1990–2000 dataset which contains clinical trial registries from 1990 to 2000. We chose only those trials that were interventional, meaning they contained the word Interventional in the study_type field. In the subset of trials selected in this way, we searched for an occurrence of drugs that are in the DrugBank_groups and NIH_Drug_Categories datasets. The data in clinical trials was not standardized, therefore, in addition to the main dictionary, we also used synonym datasets with alternative names, i.e., synonyms from DrugBank (DrugBank_synonyms) and NIH Drug Information portal (NIH_Drug_Synonyms). We additionally divided trials by the exact database and the registration date to analyse geographical distribution.

Interventions (field interventions) were then split if there was more than one in a trial. Separated interventions were then processed individually. We identified whether the interventions were present in the “DrugBank_Groups” dataset or the “DrugBank_synonyms” dataset. Then for every intervention, we identified if it was present in the “NIH_Drug_Synonyms” dataset. If the drug was identified from “NIH_Drug_Synonyms”, we then assigned a NIH category to a trial based on the “NIH_Drug_Categories” dataset. If several categories could be assigned, we included all. The drug was considered to be identified in both when it was found both in DrugBank and in NIH. Furthermore, we used the Levenshtein distance algorithm to improve precision. The data was then split into ClinicalTrials.gov and Non-ClinicalTrials.gov based on trial ID (ClinicalTrials.gov has the pattern “NCT” in every ID).

Our analysis worked according to a two-pass scheme. On the first pass (search based on the Levenshtein distance), the occurrences of the found drugs were replaced by a link to the corresponding entry in one of the drug dictionaries. On the second pass, the necessary statistics were already collected. The results of each step of processing the original ictrp_preprocessed_trials_1990–2000 were stored in a separate data set, so the whole process worked sequentially and, if necessary, could be restarted from any step. Drug analysis scripts were written in Python, using the pandas (McKinney, 2010) and numpy (Harris et al., 2020) packages. “Google Colab” was used as the operating environment.

## 3 Results

### 3.1 Overall content

We have identified a total of 689 793 trials that were registered on the ICTRP platform during the period of 1990–2020. Out of these, 478 345 trials were classified as interventional. By utilizing the DrugBank database, we were able to identify 225 857 agents, out of which 4 865 were unique. Meanwhile, the NIH DIP database identified a total of 173 473 agents, with 2 633 of them being unique drugs. The interventions that were common in both NIH DIP and DrugBank databases accounted for 165 370 agents. We found a total of 277 693 (58%) trials that were registered in ClinicalTrials.gov. By utilizing DrugBank types, we were able to identify 50 788 biotech and 175069 small molecules in both ClinicalTrials.gov and non-ClinicalTrials.gov groups. Small molecules were more prevalent in both ClinicalTrials.gov (95 353 trials) and the non-ClinicalTrials.gov group (79 716 trials), while the biotech type was mostly found in the non-ClinicalTrials.gov group with 29458 interventions. A total of 90 257 agents were classified under the DrugBank group “Approved” in all databases. Upon dividing them into different groups, both ClinicalTrials.gov and non-ClinicalTrials.gov had approximately the same number of approved agents, with a slightly higher prevalence in the latter group (47 217 in non-ClinicalTrials.gov and 43 040 in ClinicalTrials.gov). We identified 16 941 investigational and 12 240 experimental interventions in all databases. The investigational studies were more frequently found in the ClinicalTrials.gov database with 10 749 investigational agents, while the experimental studies were more prevalent in non-ClinicalTrials.gov with 8 766 experimental drugs.

**FIGURE 2:**
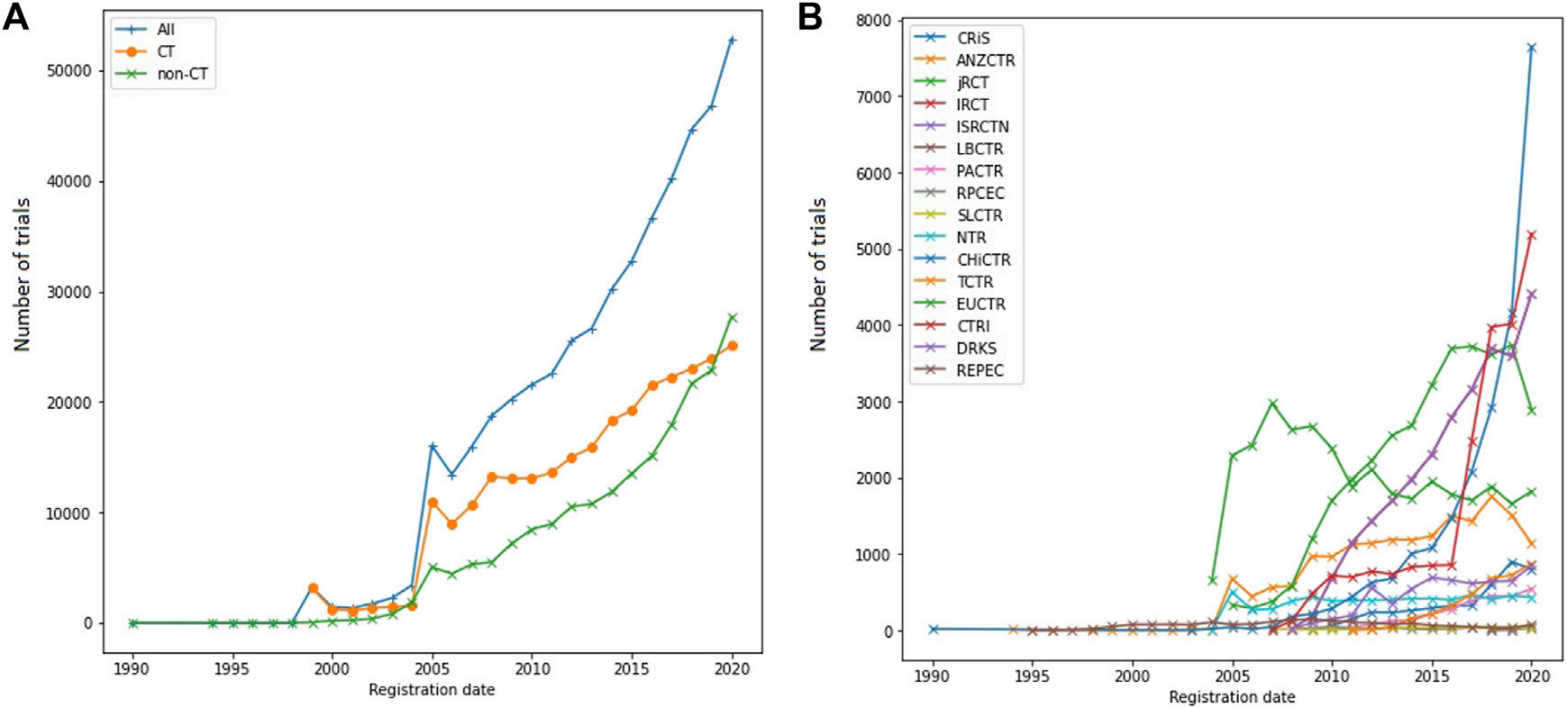
**(A)** Annual numbers of registered trials between clinicaltrials.gov and non-clinicaltrials.gov. CT—clinicaltrials.gov, non-CT—non-ClinicalTrials.gov. **(B)** Annual numbers of registered trials between separate registries in the non-clinicaltrials.gov group. Both clinicaltrials.gov and non-clinicaltrials.gov groups almost equaled by the year 2020 with a slight prevalence in the number of trials in the non-clinicaltrials.gov group. Such a result was achieved by a constant increase in trials registered in the non-clinicaltrials.gov databases that started in 2005. EUCTR (34 342 trials), IRCT (27 096 trials), CHiCTR (22 915 trials) and CTRI (21 739 trials) had the highest numbers of trials identified in the ICTRP database. The most rapid increase in the number of trials found in the ICTRP was with the CHiCTR database which almost reached 8 000 trials in 2020. Second and third place in the rate of new trial registration was found to be among CTRI and ISRCTN. Despite EUCTR having the highest number of trials in the ICTRP, we did not identify any notable increase in the number of trials. We also identified a decline in the number of trials registered in jRCT in the 2019—2020 period. CRiS—Clinical Research Information Service of Republic of Korea, ANZCTR—Australian New Zealand Clinical Trials Registry, jRCT—Japan Registry for Clinical Trials, IRCT—Iranian Registry of Clinical Trials, ISRCTN—The International Traditional Medicine Clinical Trial Registry, LBCTR—Lebanese Clinical Trials Registry, PACTR—Pan African Clinical Trials Registry, RPCEC—Cuban Public Registry of Clinical Trials, SLCTR—Sri Lanka Clinical Trials Registry, NTR—Netherlands Trial Register, CHiCTR—Chinese Clinical Trial Registry, TCTR—Thai Clinical Trials Registry, EUCTR—The EU Clinical Trials Register, CTRI—Clinical Trials Registry—India, DRKS—German Clinical Trials Register, REPEC—Peruvian Clinical Trials Registry.

We then analyzed the number of registered trials from 1990 to 2020 in clinicaltrials.gov and non-clinicaltrials.gov group (Figure 2). According to Figure 2, both non-clinical trials.gov and clinicaltrials.gov databases had similar numbers of trials registered throughout the 1990–2020 period. The highest number of registered trials was found to be in 2020 and was just below 30 000 for both groups. Among the non-clinicaltrials.gov group European Union Clinical Trials Registry (EUCTR) had the largest number of trials identified in the ICTRP (34 342). A significantly high number of trials were also found among the Clinical Trials Registry India (CTRI, 21 739), Iranian Registry of Clinical Trials (IRCT, 27 096), Chinese Clinical Trial Registry (CHiCTR, 22 915) and the International Traditional Medicine Clinical Trials Registry (ISRCTN, 16 574). CHiCTR stood out with a notable increase in trials’ registration starting from around the year 2015 and reaching its maximum in 2020 with almost 8 000 identified trials in the ICTRP. CTRI was the second most rapidly growing database starting its rise in the year 2016. Finally, the International Traditional Medicine Clinical Trials Registry (ISRCTN) registry has been increasing the number of registered clinical trials since 2009 and reaching the third most rapidly growing database in 2020. Interestingly, EUCTR, despite displaying the largest number of identified clinical trials in the ICTRP, did not show a rise and had a steady rate of clinical trial registration following a decline in the year 2006. We also identified a decrease in the number of trials in Japan Registry for Clinical Trials (jRCT) starting in 2019 and continuing till 2020.

### 3.2 Hidden duplicates in the ICTRP

**FIGURE 3:**
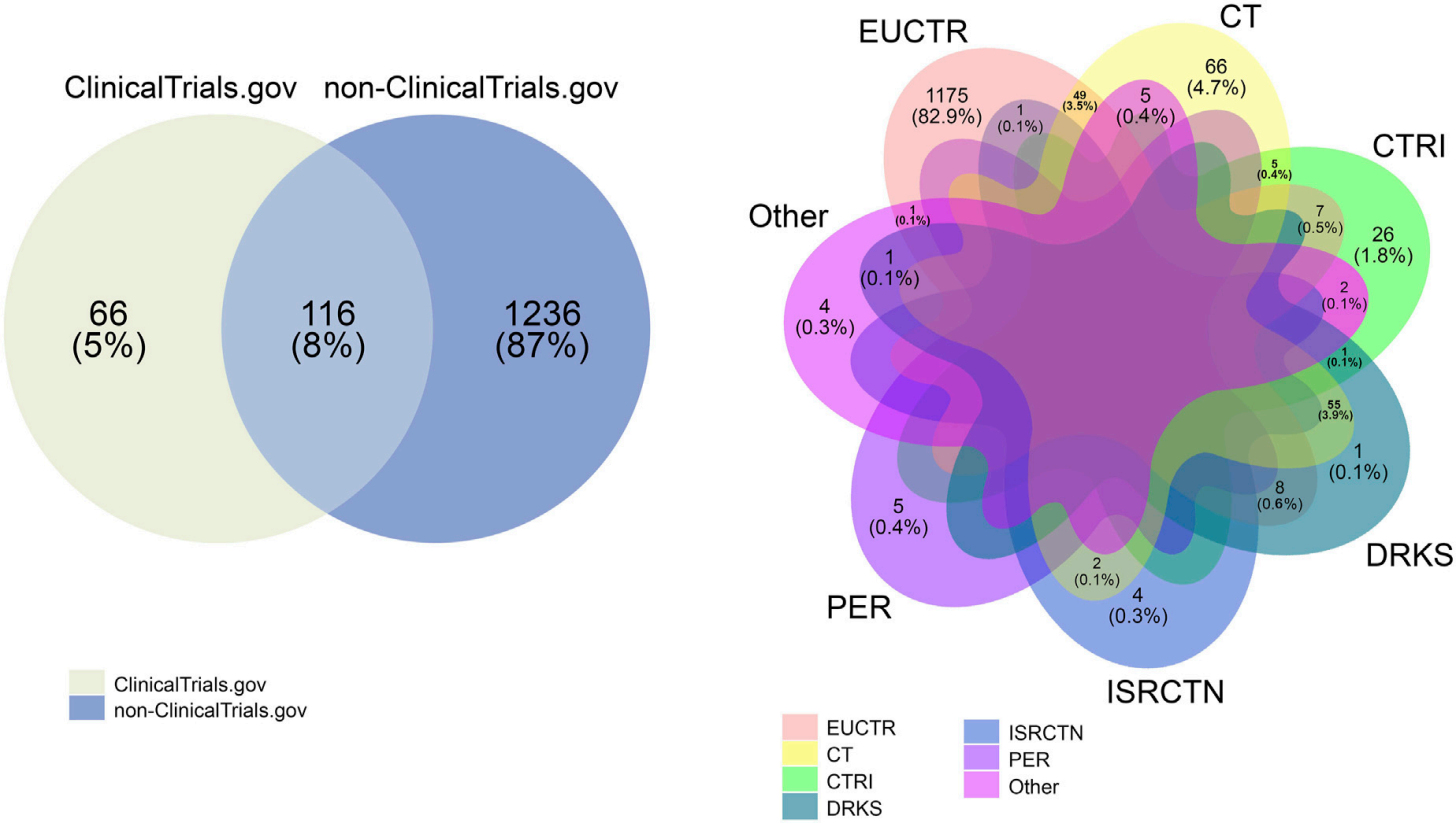
Overlapping hidden duplicate groups. An overlap between the hidden duplicates in non- 861 clinicaltrials.gov sources and clinicaltrials.gov is illustrated on the left. A more detailed view of non-clinicaltrials.gov sources is shown on the right side of this figure. As is shown in this figure, most hidden duplicates were found in the non-clinicaltrials.gov databases (87%) while clinicaltrials.gov accounted only for 5%. EUCTR had 82.9% of all hidden duplicates in the non- clinicaltrials.gov group with all other registries (CTRI, DRKS, ISRCTN and PER) ranging from 0.1% to a maximum number of 1.8% of hidden duplicates. EUCTR indicates EU Clinical Trials Register; CT, ClinicalTrials.gov; CTRI, Clinical Trials Registry—India; DRKS, German Clinical Trials Register; ISRCTN, International Standard Randomised Controlled Trial Number Registry; PER, Peruvian Clinical Trial Registry.; Other, Thai Clinical Trials Registry (TCTR), Pan African Clinical Trial Registry (PACTR), Chinese Clinical Trial Registry (CHiCTR), Japan Primary Registries Network (JPRN).

Initially, 689 793 studies were present between 1990 and 2020 in the ICTRP platform. Excluding studies without a registration date resulted in 688 866 studies. After the de-duplication process, 72842 visible duplicates were excluded resulting in 616 024 trials. The hidden duplicate algorithm resulted in the identification of 3 220 trials that have been identified as part of one of the hidden duplicate groups. In total, 1 418 hidden duplicate groups were present and 1 816 hidden duplicates were removed resulting in 614 208 trials. Figure 3 shows the overlap between the hidden duplicate groups. Looking at the overlapping groups between registries showed that 87% of all identified hidden duplicates have been studies registered in non-ClinicalTrials.gov. Only 4.7% of duplicated studies were registered exclusively in ClinicalTrials.gov, while 8% were included both in ClinicalTrials.gov and non-ClinicalTrials.gov. Out of 8% overlap between ClinicalTrials.gov and non-ClinicalTrials.gov hidden duplicates 3.5% were between ClinicalTrials.gov and 3.9% between ClinicalTrials.gov and German Clinical Trials Register. Specifically, 82.9% were within-registry duplicates coming from the EUCTR registry. All other non-ClinicalTrials.gov databases did not contain such a significant amount of duplicates with CTRI being the second largest and accounting only for 1.8% of within-registry duplicates. In total, the non-ClinicalTrials.gov duplicates were a part of 10 different registries. These results show that at least 0.5% of the entire ICTRP platform are hidden duplicates that currently go undetected.

### 3.3 Disease indications in the registries

**FIGURE 4:**
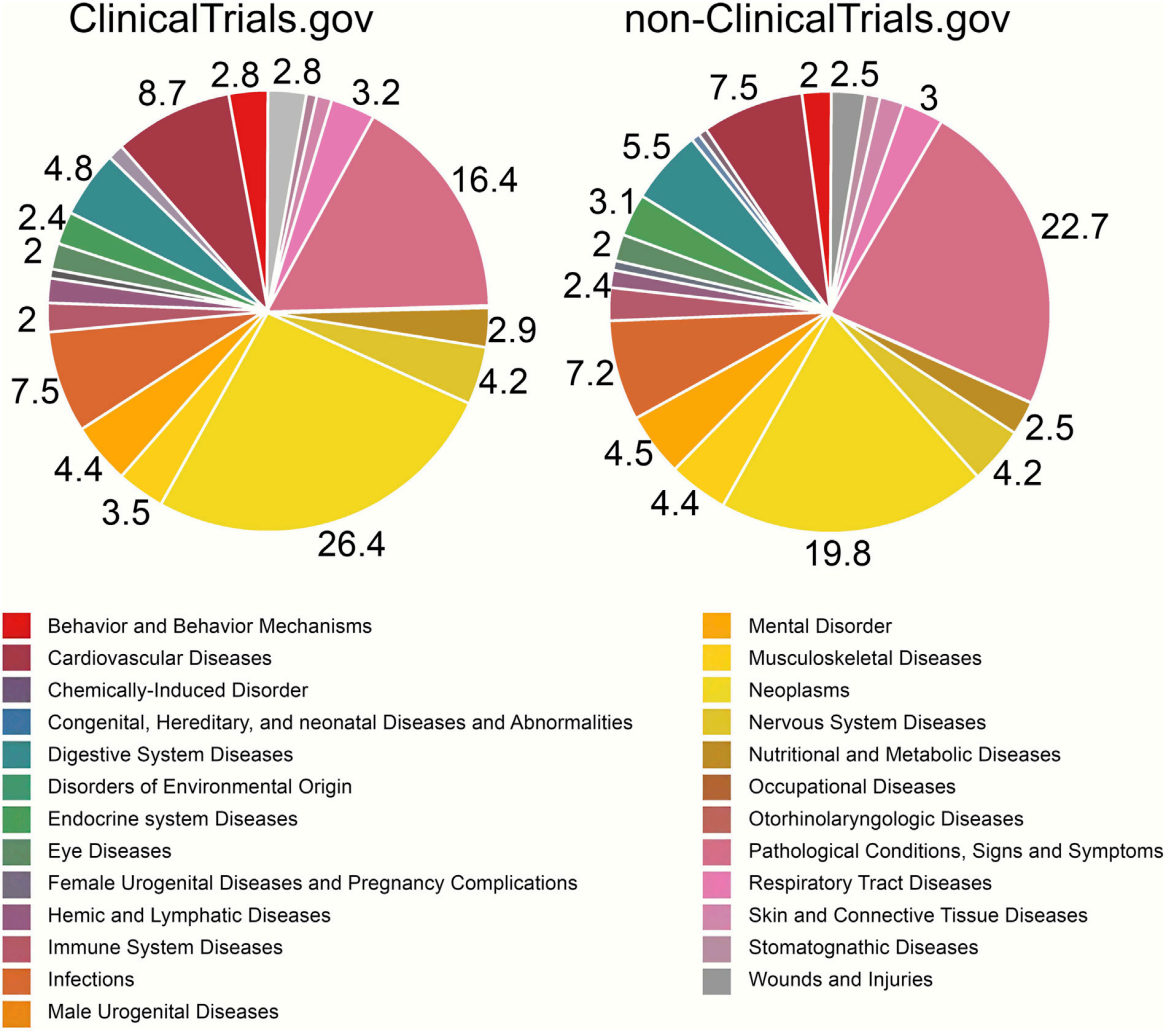
Distribution of disease classes between registry groups. The neoplasms category was one of the top conditions mentioned in trials both in clinicaltrials.gov (26.4%) and non-clinicaltrials.gov (19.8%). However, Pathological Conditions, Signs and Symptoms (22.7%) in non-clinicaltrials.gov outscored Neoplasms, while in clinicaltrials.gov Neoplasms were the leading category. Cardiovascular Diseases were in third place in both groups (clinicaltrials.gov—8.7%, non-clinicaltrials.gov—7.5%). Obtained results indicate a little difference in conditions studied in clinical trials in non-clinicaltrials.gov databases and clinicaltrials.gov. Categories with percentages below 2% are not labeled.

After the pre-processing steps, the proportions of condition categories in studies registered in ClinicalTrials.gov and non-ClinicalTrials.gov were investigated to identify the top main conditions that have been studied between 1990 and 2020. Figure 4 shows the distribution of diseases across the registries. Out of 614 208 trials, 495 247 trials have matched with a specified disease or condition. In total 4 339 conditions were present in the ICTRP platform. In ClinicalTrials.gov, the largest groups were Neoplasms (26.4%) and Cardiovascular Diseases (8.7%). Mental Disorders, containing depression trials, consisted of 4.4%. In non-ClinicalTrials.gov, the top categories were Neoplasms (19.8%) and Cardiovascular Diseases (7.5%). The search for different conditions was performed using the MESH database and we intended to imitate regular database use without any data cleansing, which also in a separation of the group named ―Pathological Conditions, Signs and Symptoms (22.7% in non-clinicaltrials.gov and 16.4% in ClinicalTrials.gov). The results show that in total the order of disease classes and percentages of certain categories were similar between ClinicalTrials.gov and non-ClinicalTrials.gov. 304.

### 3.4 Identification of words in the intervention section in clinical trials using DrugBank database

A total of 478 345 clinical trials from both ClinicalTrials.gov and non-clinicaltrials.gov were analyzed. Our analysis was based on words matching in the Intervention section and in DrugBank or NIH DIP. We did not manually exclude any matched words that did not fall under category of drug to achieve unbiased results on databases content and application in research. The below described results contain both drugs and other words identified in the Intervention section. Among 478 345 clinical trials, 219 372 interventions did not include any drugs/words identified by DrugBank or NIH DIP databases. Some examples of such interventions included physical exercise, blood withdrawal or wearing contact lenses. Using DrugBank we identified 4 865 unique drugs/words, NIH DIP database detected 2 633 unique drugs/words.

**FIGURE 5:**
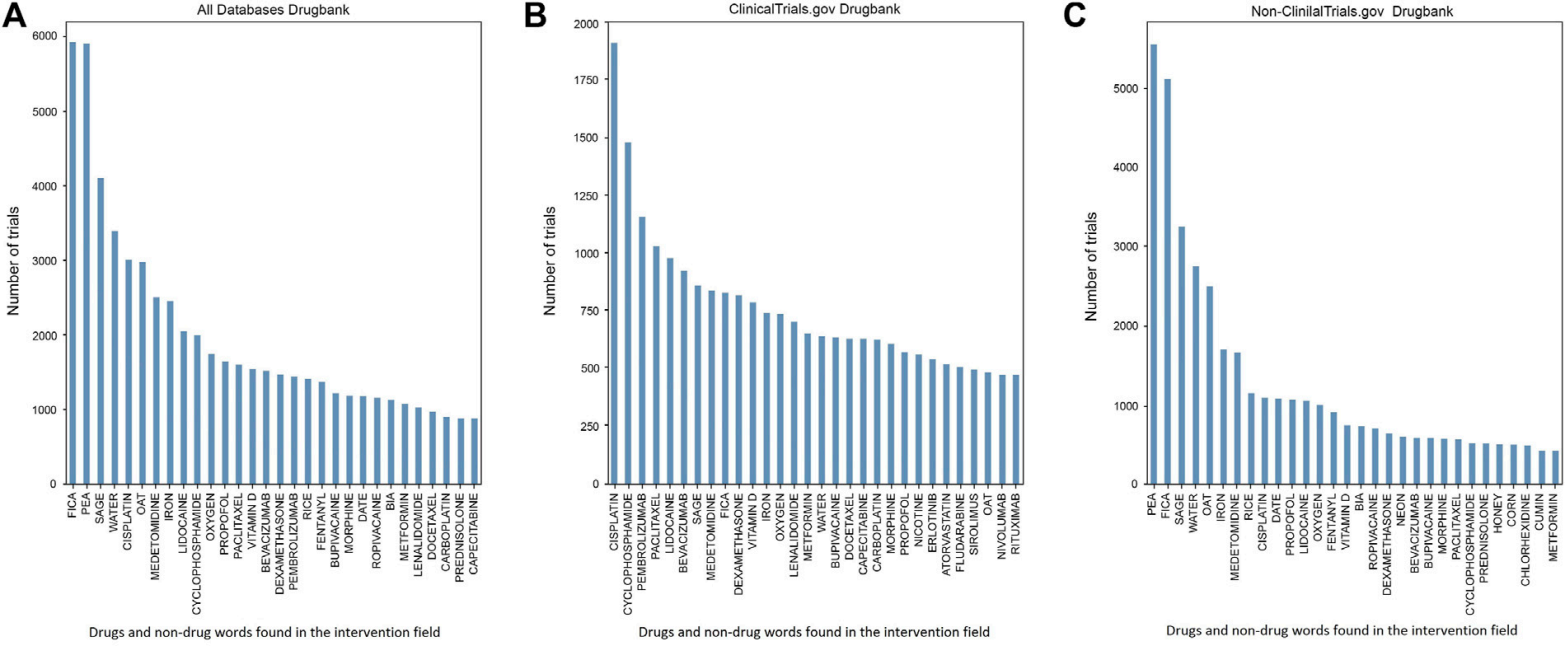
**(A)** Top identified drugs and non-drug words in the Intervention field using DrugBank in clinicaltrials.gov and non-clinicaltrials.gov groups. **(B)** Top identified drugs and non-drug words in the Intervention field using DrugBank in clinicaltrials.gov database. **(C)** Top identified drugs and non-drug words in the Intervention field using DrugBank in non-clinicaltrials.gov databases. We identified a high number of non-drug words in the non-clinicaltrials.gov group including pea (5 537 trials), fica (5 083 trials), sage (3 242 trials), oat (2 489 trials), water (2 746 trials), rice (1 151 trials), date (1 090 trials), neon (612 trials), honey (512 trials) and corn (508 trials). These words were found in the Intervention field but also marked as drugs in the DrugBank database. The non-drug words were also present among the clinicaltrials.gov database but in smaller number and variety: sage (859 trials), fica (825 trials), oat (480 trials) and water (637 trials). Cisplatin was the drug with most trials in the clinicaltrials.gov group (1 908 trials). Widely represented categories of drugs in both **(B,C)** were anticancer therapy (cisplatin, cyclophosphamide, paclitaxel and bevacizumab) and anesthetics and analgesics (bupivacaine, ropivacaine, propofol, lidocaine and medetomidine). Among anticancer therapy, in the clinicaltrials.gov group, a notable part was dedicated to monoclonal antibodies (pembrolizumab with 1 153 trials or bevacizumab with 924 trials). Vitamin D and iron were present in both clinicaltrials.gov (783 trials) and non-clinicaltrials.gov (757 trials) groups.

First, we identified various words in clinical trials using the DrugBank database as a source for drug names. We then divided them into two groups: words from the ClinicalTrials.gov database and words from any other databases which were named Non-clinicaltrials.gov. Our results are illustrated in Figure 5. ClinicalTrials.gov has many studies on anticancer therapy. Cisplatin, for instance, accounted for almost 2 000 clinical trials (1 908 trials), paclitaxel exceeded 1 000 trials (1 029 trials) and cyclophosphamide appeared in nearly 1 500 instances (1 477 trials). An alternative platinum-based chemotherapy drug was carboplatin (more than 500 trials). Among anticancer therapy, a notable number of trials were dedicated to monoclonal antibodies. For example, pembrolizumab, a drug used as an immune checkpoint inhibitor was the third most commonly encountered drug. Another frequently applied antibody was bevacizumab, designed to bind vascular endothelial growth factor (VEGF) and often used in combination with other antitumor therapies. According to our results, the second most abundantly represented group was anesthetics and analgesics. Within this group, lidocaine was the most studied drug (980 trials). Other mentioned drugs identified by DrugBank were: bupivacaine (634 trials), propofol (570 trials), and medetomidine (838 trials). ClinicalTrials.gov interventions section also contained words that could not be attributed to drugs such as sage (859 trials), fica (825 trials), water (637 trials), oat (480 trials) and iron (738 trials).

The identified order of words among the non-clinicaltrials.gov interventions group differed from those in the clinicaltrials.gov database. A greater number of non-drug words were identified in the intervention sections. In the non-clinicaltrials.gov group we identified the following words: neon (612 trials), date (1 090 trials), pea (5 537 trials), fica (3 083 trials), sage (3 242 trials), water (2 746 trials), oat (2 489 trials), iron (1 709 trials), rice (1 151 trials), honey (512 trials), corn (508 trials) and cumin (434 trials). For instance, in the clinicaltrials.gov group sage was present in 859 trials while in the non-clinicaltrials.gov the result was 3 242 trials making it the third most commonly encountered word in this group. In non-clinicaltrials.gov databases pea turned out to be the most frequent word marked as an intervention both by clinical trials databases and DrugBank (5 537 trials). However, the general concept of prevailing anticancer treatment seen in the clinicaltrials.gov group was still present. Such drugs as cisplatin, bevacizumab, paclitaxel and cyclophosphamide were identified in the non-clinicaltrials.gov group, while they were not as frequently studied as in clinicaltrials.gov database (cisplatin being the number one studied drug with 1 908 trials in clinicaltrials.gov and reaching only 1 091 trials in non-clinicaltrials.gov group). The anesthetics and analgesics group comprised more drugs and trials than in clinicaltrials.gov with medetomidine (1 668 trials), propofol (1 073 trials), lidocaine (1 060 trials), ropivacaine (711 trials), fentanyl (915 trials) and bupivacaine (589 trials).

### 3.5 Identification of words in the intervention section and drug categories in clinical trials using NIH DIP database

**FIGURE 6:**
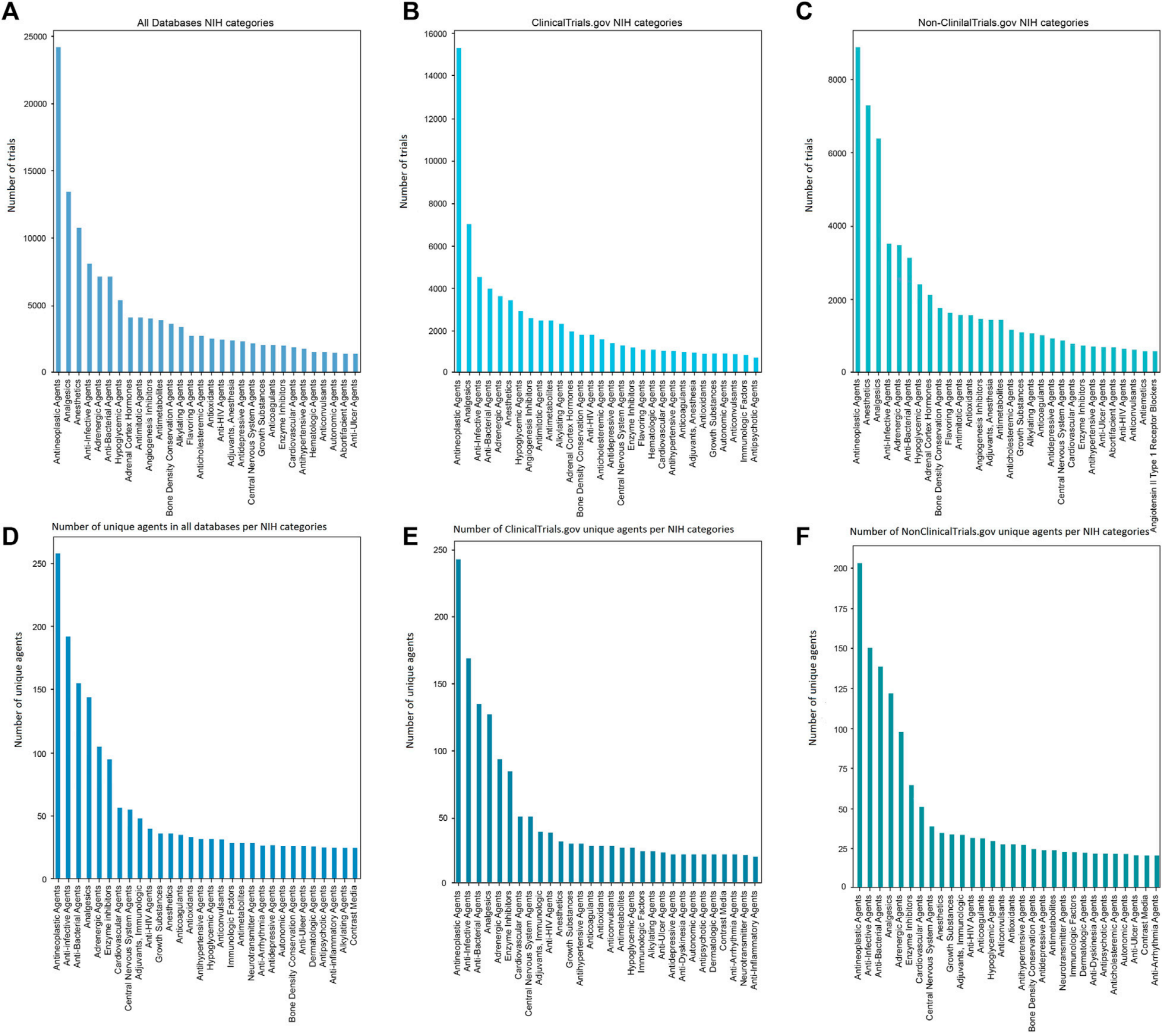
NIH categories in **(A)** all databases, **(B)** clinicaltrials.gov and **(C)** non-clinicaltrials.gov databases. Unique NIH categories are represented in **(D)** all databases, **(E)** clinicaltrials.gov, **(F)** non-clinicaltrials.gov databases. Antineoplastics agents were widely represented in clinicaltrials.gov group with 15 309 trials while reaching only 8 873 registries in the non-clinicaltrials.gov group. Both groups had approximately the same number of unique agents with 245 unique antineoplastics clinicaltrials.gov drugs and 203 non-clinicaltrials.gov medications. Non-clinicaltrials.gov group comprised a large number of anesthetics (7 303 trials compared to 3 428 trials in clinicaltrials.gov). However, both groups differed only by 3 unique agents with clinicaltrials.gov having 32 drugs and non-clinicaltrials.gov having 35 unique drugs. In clinicaltrials.gov, anti-infective (4 568 trials) and anti-bacterial agents (3 979 trials) also played a definitive role. Such categories as antidepressive agents (924 trials in non-clinicaltrials.gov and 1 420 trials in clinicaltrials.gov), cardiovascular agents (788 trials in non-clinicaltrials.gov and 1 066 trials in clinicaltrials.gov) or central nervous system agents (864 trials in non-clinicaltrials.gov and 1 294 trials in clinicaltrials.gov) appeared to be slightly underrepresented in comparison to other categories with most trials being identified in clinicaltrials.gov database.

Next, the NIH DIP database was used to identify top drug categories in clinicaltrials.gov and non-clinicaltrials.gov groups (Figure 6). Antineoplastic agents were present in the most number of trials in all databases with 15 309 trials identified in clinicalrials.gov and 8 873 in non-clinicaltrials.gov. This category also stood out in the number of unique agents with 243 unique agents in clinicaltrials.gov and 203 in nonclinicaltrials.gov. In clinicaltrials.gov other large groups were anti-infective and anti-bacterial agents reaching 8 547 trials in total. Unique anti-infective agents were identified in 169 trials while anti-bacterial drugs were slightly less common with 135 agents found. Analgesics maintained to be in the top categories with 127 unique drugs and 7 013 in total. However, anesthetic category was found to be not so diverse with only 32 unique agents. Cardiovascular agents (1 294 trials), central nervous system agents (1 294 trials) and enzyme inhibitors (1 243 trials) had a remarkable amount of unique agents given a relatively small number of trials with 51 unique drugs in the central nervous system and cardiovascular categories each and 85 in enzyme inhibitors.

In the non-clinicaltrials.gov databases the trends for top categories were consistent with the results achieved for clinicaltrials.gov. Anesthetics (7 303 trials) and analgesics (6 400 trials) occupied second and third place respectively by the total number of clinical trials. Despite anesthetics category exceeding the number of trials devoted in clinicaltrials.gov by almost 4 000, the total number of unique agents was found to be approximately the same (35 unique drugs in non-clinicaltrials.gov and 32 in clinicaltrials.gov). Anti-infective and antibacterial agents also played a notable role in non-clinicaltrials.gov databases with 3 515 and 3 133 trials, respectively. Adrenergic agents were the fifth most common category with 3 487 total trials and 98 unique trials. Two categories not present in clinicaltrials.gov top were abortifacient agents with 692 trials and flavoring agents with 1 646 clinical trials identified. These categories, however, were not present in top unique agents, denoting that the exact number of unique drugs is less than 21. In turn, we also identified categories which were present only in top unique agents indicating a small number of trials but a wide range of the exact drugs. Contrast media and anti-arrhythmia agents had 21 unique trials each. Immunologic factors and dermatologic agents were present in 23 unique clinical trials.

The first eight unique categories were the same in all registries with enzyme inhibitors, cardiovascular agents and central nervous system agents present in the top ten categories in contrast to (b) and (c) in Figure 6 where these categories appeared to be scarce (1 066 trials with cardiovascular agents in clinicaltrials.gov and 788 in the non-clinicaltrials.gov databases). However, adjuvants were only present in unique clinicaltrials.gov categories with 34 trials. The same could be stated for anti-inflammatory agents absent in non-clinicaltrials.gov databases and having 21 trials with unique drugs. All the other categories showed very small differences in the numbers of unique agents. For instance, hypoglycemic agents in non-clinicaltrials.gov had 30 unique agents and in clinicaltrials.gov this number was 28.

**FIGURE 7:**
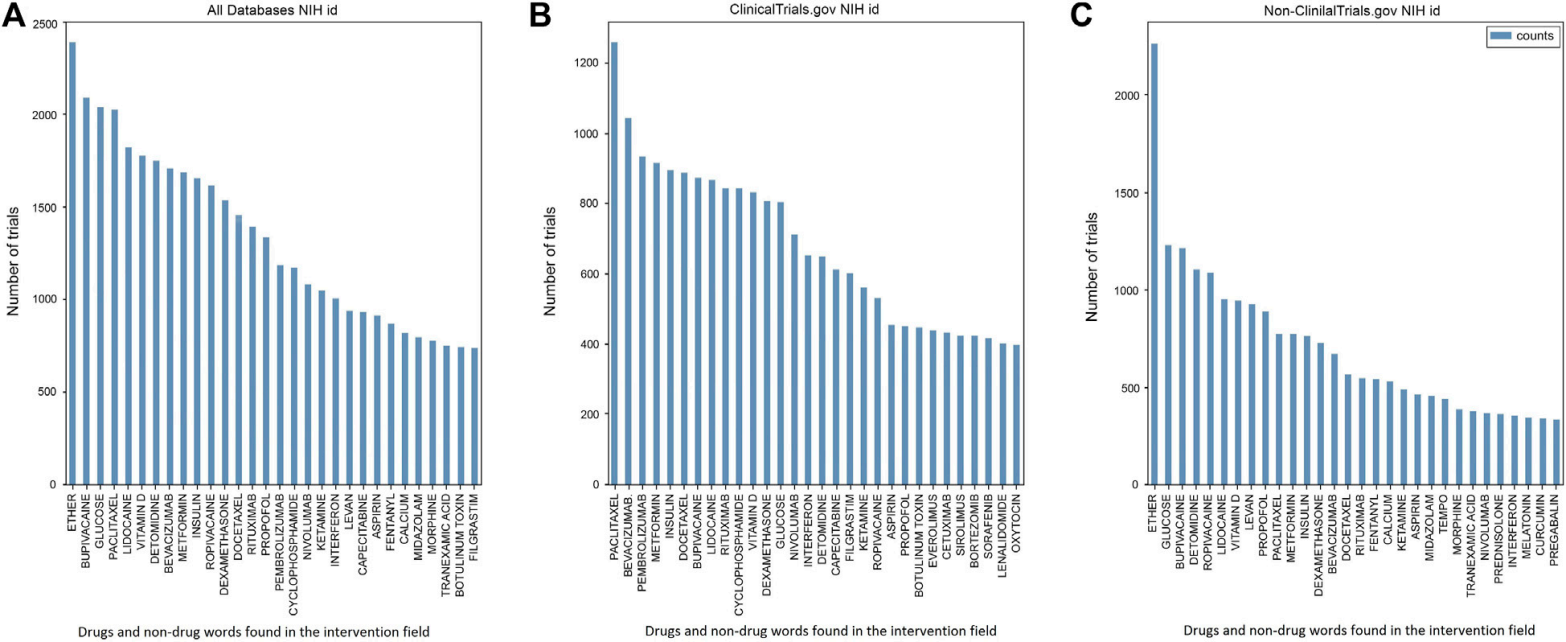
Drugs and non-drug words identified in the Intervention field using NIH id in **(A)** both groups, **(B)** clinicaltrials.gov database and **(C)** non-clinicaltrials.gov databases. Vitamin D was consistently present in all databases with 949 trials in non-clinicaltrials.gov and 829 in clinicaltrials.gov. Clinicaltrials.gov’s top drugs were either anticancer therapy [paclitaxel (1 256 915 trials), pembrolizumab (932 trials), docetaxel (888 trials) and bevacizumab (1 042 trials)] or antidiabetics with 915 trials on metformin and 893 on insulin. Anesthetics and analgesics were one of the most abundant groups in non-clinicaltrials.gov databases comprising ether (2 265 trials), lidocaine (954 trials), ropivacaine (1 087 trials), propofol (885 trials), fentanyl (542 trials), ketamine (486 trials) and morphine (383 trials). The two most commonly studied drugs among the non-clinicaltrials.gov group were ether (2 265 trials) and glucose (1 232 trials). In contrast to DrugBank, we did not identify any non-drug words in the clinicaltrials.gov top drugs using NIH. Non-drug words were present in the non-clinicaltrials.gov database but were notably smaller in variety and numbers of trials than in the same group in DrugBank. Among non-drug words we identified levan (923 trials), tempo (441 trials) and curcumin (338 trials).

The NIH DIP id was used as a source for drug names and other words found in the intervention section (Figure 7). In the clinicaltrials.gov database two most abundant groups were anticancer therapy and antidiabetics. Among the first group, most clinical trials contained paclitaxel (1 256 trials), bevacizumab (1 042 trials) and pembrolizumab (932 trials). The antidiabetic group was slightly less common than anticancer drugs primarily comprising metformin with 915 trials and insulin with 893 trials. In contrast to DrugBank anticancer group in clinicaltrials.gov had fewer monoclonal antibodies with rituximab and involumab being absent in NIH DIP top drugs. The distribution of anesthetics and analgesics also changed in comparison to DrugBank. In clinicaltrials.gov lidocaine was now identified in 866 trials, bupivacaine in 873 trials, ropivacaine in 530 trials, propofol in 450 trials and medetomidine was absent in comparison with DrugBank. However, detomidine now appeared in the top NIH DIP-identified drugs with 646 trials. The same drug was identified in a larger number of trials in non-clinicaltrials.gov databases (1 104 trials). In general, the anesthetics and analgesics group in non-clinicaltrials.gov databases was much wider represented comprising ropivacaine (1 087 trials), lidocaine (954 trials), propofol (885 trials), fentanyl (542 trials), ketamine (486 trials) and morphine (383 trials). The most common drug in non-clinicaltrials.gov was ether (2 265 trials) with glucose coming second reaching 1 232 trials. In contrast to clinicaltrials.gov, anticancer therapy was not as common compared to, for instance, anesthetics and analgesics. Paclitaxel was found only in 770 trials, bevacizumab in 667 trials, docetaxel and rituximab in 564 and 550 trials respectively.

In contrast to DrugBank, we did not identify any non-drug words in the first 30 clinicaltrials.gov interventions. However, the same could not be applied to the non-clinicaltrials.gov database. The NIH DIP id identified such words as tempo (441 trials), curcumin (338 trials), calcium (532 trials) and levan (923 trials). Midazolam (455 trials), pregabalin (332 trials) and melatonin (348 trials) were only present in the top non-clinicaltrials.gov words found in the intervention section. Vitamin D was present in all groups with approximately the same number of trials devoted (949 trials in non-clinicaltrials.gov and 829 in clinicaltrials.gov).

### 3.6 Comparison of DrugBank and NIH DIP databases

**FIGURE 8:**
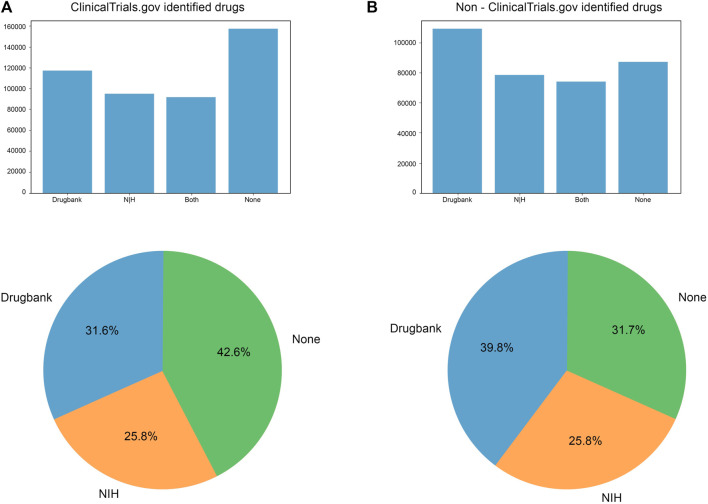
Trials identified using NIH id and DrugBank in **(A)** clinicaltrials.gov and **(B)** non-clinicaltrials.gov group. DrugBank identified more trials than NIH id in both groups (31.6% in clinicaltrials.gov and 39.8% in non-clinicaltrials.gov). Both NIH (28.5%) and DrugBank (39.8%) showed better performance among non-clinicaltrials.gov registries (in comparison to 31.6% DrugBank and 25.8% NIH in the clinicaltrials.gov group). Therefore, more trials were found in non-clinicaltrials.gov registries with the None category being remarkably reduced compared to the clinicaltrials.gov group (from 42.6% to 31.7%). In terms of exact number, clinicaltrials.gov group contained more than 140 000 not identified trials, while non-clinicaltrials.gov did not reach 100 000.

Given the difference between words identified by DrugBank and NIH DIP id, we performed a quantitative comparison of registries with respect to databases to deduce which database was most sufficient to match the words in the intervention section of clinical trials (Figure 8). DrugBank performed approximately the same in both clinical trial groups with a slight skew towards non-clinicaltrials.gov group (31.6% for clinicaltrials.gov and 39.8% for non-clinicaltrials.gov). DrugBank was able to identify more words than NIH DIP dataset in both groups. NIH DIP database presented roughly the same results with 25.8% in clinicaltrials.gov and 28.5% in non-clinicaltrials.gov. It must be noted that both DrugBank and NIH DIP could not identify more than half of the interventions applied in clinical trials. Most of the unidentified trials were found to be in clinicaltrials.gov with almost 160 000 of them undetected (42.6%). In contrast, non-clinicaltrials.gov had much less than 100 000 unidentified trials which accounted for 31.7%. A decrease in undetected words could be explained by a better performance of DrugBank in the non-clinicaltrials.gov group.

## 4 Discussion

### 4.1 Overall characteristics of the ICTRP platform

The ICTRP is an expansive global platform designed to offer a vast and easily accessible repository of clinical trial registries from across the world. Our search process yielded 689 793 trials spanning from 1990 to 2020, obtained from 17 diverse registries. In contrast, ClinicalTrials.gov, the commonly used registry, had fewer studies or 362 500 studies registered as of 2020, underscoring the fact that ICTRP contains important data beyond the ClinicalTrials.gov. Our analysis disclosed that EUCTR had the largest number of identified trials, with a total of 34 342 registered through ICTRP (as demonstrated in Figure 2). EUCTR has remained relatively stable in recent years, following a steady pattern after a decline in 2006, with no significant increase in its number of registered trials. However, a recent review identified issues with data availability in the EUCTR, such as the absence of protocols for 66% of studies from Norway, France, and Poland (DeVito and Goldacre, 2022). Additionally, another review noted discrepancies in completion status between the EUCTR and clinicaltrials.gov databases (Fleminger and Goldacre, 2018). To address this concern, retrospective registration in the EUCTR database was no longer permitted (Goldacre et al., 2018). Notably, Iran’s IRCT was among the leading countries with the highest number of newly registered trials in non-clinicaltrials.gov, presenting 27 096 trials recorded in the ICTRP database (Figure 2). In 2009, a review identified IRCT as WHO’s primary register (Solaymani-Dodaran et al., 2009). Since then, other reviews have noted a substantial and continuous rise in the number of registered trials due to well-implemented registration policies (Solaymani-Dodaran et al., 2011; Ali et al., 2019). However, some authors have expressed concerns about retrospective registration and the concentration on diseases that make up a small proportion of the total disease burden in Iran (Solaymani-Dodaran et al., 2013; Feizabadi et al., 2017). A recent review has shown a high publication rate for trials registered in the IRCT (Lofti et al., 2020). Simultaneously, there’s been a continuous expansion in the scope of clinical trials. In 2020, both Iran and China emerged as leaders in clinical trials involving probiotics (Dronkers et al., 2020). We observed that clinical trials registered in the IRCT are underrepresented in reviews when compared to those registered in ClinicalTrials.gov. Concerns have also been raised about CTRI and CHiCTR, particularly regarding their retrospective registration policies (Birajdar et al., 2019; Zhang et al., 2019). However, our findings indicate a consistent increase in the registration of non-ClinicalTrials.gov intervention trials. This trend aligns with observations made by other researchers, as seen in the work of Banno et al. (2019), highlighting the growing significance of this data source. Nonetheless, it remains crucial to acknowledge both the strengths and limitations of the ICTRP database to ensure that our results are unbiased.

### 4.2 The analytical integrity of the platform

Here we proposed a detection and elimination algorithm for the presence of duplicate registrations or so called hidden duplicates (Saberwal, 2021). Our analysis revealed that 0.5% of all trials in the ICTRP remained undetected hidden duplicates. This percentage marks a significant decline compared to the 5% detected in a 2016 paper (van Valkenhoef et al., 2016). The vast majority of these hidden duplicates were found in the EUCTR database (82.9%), while ClinicalTrials.gov had a much lower number of within-registries duplicates (4.7%). Other databases had relatively small numbers of hidden duplicates, with CTRI having the largest at 1.8%. Interestingly, the largest overlap between databases was found between DRKS and ClinicalTrials.gov; together, these databases contained 3.9% of the hidden duplicates found in both databases (Figure 3). Over the years, ICTRP has continuously introduced new strategies to identify duplicate trials, such as the “prospective registration” field. These measures have contributed to significant improvements in the detection of duplicate trials.

During our analysis of clinical trial data, we faced a significant challenge related to sections that contained information on interventions and drugs used in these trials. To assess trends in specific drugs, we developed a program based on word matching between the intervention section and DrugBank or NIH DIP. However, the results we obtained were surprising, particularly for non-ClinicalTrials.gov databases. For instance, DrugBank often identified words unrelated to drugs or interventional trials, such as “date,” “sage,” “corn,” “pea,” “fica,” and “honey.” Although NIH DIP identified a smaller number of non-drug words, we still noted terms like “tempo,” “calcium,” and “levan” in non-ClinicalTrials.gov databases. Interestingly, NIH DIP did not identify any non-drug words in the top interventions on clinicaltrials.gov. Some of these words could be classified as nutraceuticals, but others like “date” and “tempo” could not be attributed to any such category. Thus, to perform an all-encompassing analysis of drug trends, we have two potential options. Firstly, we could apply more sophisticated drug recognition techniques that require advanced programming, or secondly, we could engage in a manual curation or “cleaning” process of the ICTRP, DrugBank, and NIH DIP (Piliouras et al., 2013; Javed et al., 2021). However, this is a complex task that requires significant effort. It is notable that while comparing ClinicalTrials.gov and the ICTRP platform, ClinicalTrials.gov stands out as having a more automated system of data entry, whereas the ICTRP remains dependent on a less structured and standardized system. This difference is largely due to the varying standards in the initial registries. Standardization of fields like specified conditions and interventions, as well as improvements in duplicate identification, could significantly enhance the platform (van Valkenhoef et al., 2016; Kumari et al., 2020; Saberwal, 2021; Thiele et al., 2021).

### 4.3 Trends in disease indications

Our analysis of disease trends across different clinical trial registries (as seen in Figure 4) shows considerable consistency. In ClinicalTrials.gov, we found that neoplasms were the largest category of trials, followed by cardiovascular diseases, infectious diseases, digestive system diseases, and mental disorders. A previous oncology systematic analysis observed a similar distribution between 2007 and 2010, with oncology being the largest category, followed by mental health, infectious diseases, diabetes mellitus, and cardiology (Hirsch et al., 2013). The proportions of non-ClinicalTrials.gov were similar, with the highest categories being neoplasms, cardiovascular diseases, infectious diseases, digestive diseases, and mental disorders. These results suggest that prioritization of researching certain disorders is similar between US-registered studies and studies listed elsewhere. Ongoing research in neoplasms continues to drive a surge in investigational agents and clinical trials, with immunotherapy using monoclonal antibodies for cancer treatment representing a specific area of extensive research (Kruger et al., 2019; Filin et al., 2020; Majeed et al., 2021; Moreno-Cortes et al., 2021). Though generally encouraging, the results of CAR-T and other immunotherapy therapies have certain limitations in treating solid tumors, as previously noted by the authors (Filin et al., 2020). Additionally, checkpoint inhibitors are emerging as another preferred method for the treatment of neoplasms, further contributing to the prevalence of neoplasms in both ClinicalTrials.gov and non-ClinicalTrials.gov databases (26.4% and 19.8%, respectively) (Kruger et al., 2019). Cardiovascular diseases accounted for the second-largest category in both groups. Cardiovascular diseases accounted for the second-largest category in both groups. Although global mortality rates from cardiovascular diseases have declined in recent years, the global burden remains significant (Jagannathan et al., 2019; Amini et al., 2021). An observational study highlighted the slow drug approval process for conditions like obesity, atherosclerosis, and diabetes, which are common conditions associated with cardiovascular diseases (Batta et al., 2020). Additionally, between 1990 and 2012, the number of cardiovascular agents entering clinical trials decreased (Hwang et al., 2016). Currently, dyslipidemia drugs represent a substantial part of clinical cardiovascular research, with the emerging PCSK9 antibodies and antisense oligonucleotides further boosting research in this area (Jang et al., 2021).

### 4.4 Common drugs and drug categories in clinical trials

Our analysis revealed a predominance of antineoplastic therapy across all databases, with a notable higher numbers in the clinicaltrials.gov with 15 309 trials compared to non-clinicaltrials.gov with only 8 873 trials. However, the number of unique agents represented in these trials did not exceed 250 in all databases, accounting for only 1.6% of all trials in the clinicaltrials.gov group (as seen in Figure 6). A 2017 study using an advanced dynamic topic model noted a downward trend in antineoplastic agents (Anand et al., 2017). The same analysis also found evidence of an upward trend within the cardiovascular disease group, which was not directly evident from our results. Most cardiovascular trials were registered in the clinicaltrials.gov group (1 066 trials), although the number of unique agents was relatively low compared to antineoplastic treatment (50 unique agents in the cardiovascular disease group, 4.7%). One possible issue within development of unique cardiovascular agents could be the discrepancies between trial outcomes and published results. This can lead to biases in the data analysis as well as problems in the actual drug research, which can impede the success of clinical trials as the actual efficacy of a drug is not well established (Hartung et al., 2014; Steinberg et al., 2022).

Anesthetics and analgesics were one of the most abundant group trialed primarily in non-clinicaltrials.gov, with a total of 7 303 trials and a wide range of tested drugs (as seen in Figure 6). The most common drug in the non-clinicaltrials.gov group was a general anesthetic or a solvent ether, with 2 265 trials (Figure 7) (Brown et al., 2018). The predominance of anesthetics and analgesics could be attributed to advancements in drug forms and the subsequent development of liposomal formulations of, for instance, bupivacaine (Prabhakar et al., 2019). It has also been found that local anesthetics interact with many other receptors aside from sodium channels, such as two-pore domain K+ channels (Gruss et al., 2004; Shah et al., 2018). This discovery has led to new drug qualities and features that require further evaluation (Kim et al., 2018). There is also an ongoing debate concerning whether certain anesthetic agents can reduce the recurrence or metastasis rates after cancer surgery (Buggy and Wall, 2019; Grandhi and Perona, 2020; Longhini et al., 2020). The abundance of unexplored effects in the anesthetics and analgesics group has led to a rise in the number of clinical trials; however, the actual number of unique agents remains low, at less than 40 agents across all databases.

NIH DIP and DrugBank could not detect a considerable number of trials out of all trials processed from the ICTRP. The highest number of undetected trials and, consequently, interventions was found in the clinicaltrials.gov group (42.6% compared to 31.7% in the non-clinicaltrials.gov group) (as seen in Figure 8). Several analysis have noted that while the clinicaltrials.gov database is convenient for accessing data, it is difficult to manage and not suitable for large scale computational analysis (Tasneem et al., 2012; Cepeda et al., 2013). As a result, before conducting any quantitative analysis of clinicaltrials.gov data, the data may have to be first converted into an appropriate format, which can be a challenging task that can hinder the analysis process. It is important to note that standardization is not only necessary for the clinicaltrials.gov database but also for the DrugBank and NIH DIP databases. However, it should be noted that the NIH DIP has been discontinued and is no longer available as of December 2022, and thus its data may have changed (National Library of Medicine, 2022).

## 5 Conclusion

The International Clinical Trials Registry Platform (ICTRP) is a rapidly expanding database. Nearly half of the trials in the ICTRP originated from Clinicaltrials.gov. The European Union Clinical Trials Registry (EUCTR) contributed the largest number of trials to the non-Clinicaltrials.gov group, with 34 342 trials identified in the ICTRP. Other notable contributors include the Clinical Trials Registry India (CTRI), with 21 739 trials, the Iranian Registry of Clinical Trials (IRCT), with 27 096 trials, the Chinese Clinical Trial Registry (CHiCTR), with 22 915 trials, and the International Traditional Medicine Clinical Trials Registry (ISRCTN), with 16 574 trials. CHiCTR in particular demonstrated a significant increase in trial registrations starting in 2015, with nearly 8 000 trials identified in the ICTRP by 2020. Similarly, CTRI has shown rapid growth since 2016. Of the total trials identified in the ICTRP, 277 693 (58%) were registered in ClinicalTrials.gov.

Regardless of registry type, the largest categories of diseases included neoplasms, cardiovascular diseases, infectious diseases, digestive system diseases, and mental disorders. In ClinicalTrials.gov, neoplasms (26.4%) and cardiovascular disorders (8.7%) were the most commonly studied diseases. This trend was also observed in non-ClinicalTrials.gov databases, with neoplasms accounting for 19.8% and cardiovascular disorders accounting for 7.5% of trials. These findings suggest that ClinicalTrials.gov and non-ClinicalTrials.gov databases have similar priorities for disease research. Antineoplastic therapy was the most prevalent drug category in ClinicalTrials.gov, with over 15 000 drugs studied. In the non-ClinicalTrials.gov databases, anesthetics and analgesics showed promising growth with 7 303 drug trials. In general, non-ClinicalTrials.gov databases demonstrated higher number of anesthetics and analgesic drug studies, with a wider range of studied drugs. The number of unique interventions was relatively low, with only 250 unique agents identified in the antineoplastic therapy category. This suggests a lack of newly registered therapies, which may stem from inconsistencies between preclinical and clinical studies, as has been previously suggested in the literature.

Our study reveals a notable reduction in the overall number of hidden duplicates, which highlights the effectiveness of our relatively simple method for analyzing big datasets, preprocessing and finding/removing duplicates (including hidden ones). While the intervention field had several highly frequent non-drug words such as peas, date, and tempo, our method allowed us to identify those gaps and mitigate them. ICTRP accounted for nearly 0.5% of hidden duplicates, previously undetected by the system. The Non-clinicalTrials.gov group contained 87% of duplicates with 82.9% found in the EUCTR database. CTRI, IRCT, and CHiCTR were other databases with a high number of identified trials but had a minimal contribution to hidden duplicates. It is worth noting that the percentage of hidden duplicates is much smaller (less than 5%) than in the previous 2016 study (van Valkenhoef et al., 2016), indicating significant improvement in the deduplication process across most databases, except for the EUCTR. In the ICTRP intervention fields, we identified many non-drug words—such as “peas” and “dates”—without any standardized labeling, creating significant obstacles during analysis. Additionally, while DrugBank and NIH DIP treated these words as drugs on par with actual agents, there was a lack of uniformity in the data, which required more sophisticated programming methods for analysis. Our analysis revealed that DrugBank performed better than NIH DIP, identifying most trials in non-ClinicalTrials.gov databases (39.8%). However, both databases experienced difficulty with identifying agents due to the presence of non-drug words. Further analysis showed that DrugBank had the greatest number of non-drug words among non-ClinicalTrials.gov groups, while NIH DIP exhibited better performance in the ClinicalTrials.gov group and displayed no non-drug words. To facilitate a thorough analysis of clinical trial trends, standardization of data across various databases is essential. We therefore propose that DrugBank and the ICTRP would benefit from data reorganization and cleansing to enhance their performance. While the ICTRP is a highly valuable resource for large-scale medical analysis, it is critical to acknowledge the limitations of the data, including its strengths and weaknesses, to ensure substantial curation before automating large-scale analysis of medical trends.

## Data Availability

Publicly available datasets were analyzed in this study. This data can be found here: https://www.who.int/clinical-trials-registry-platform https://clinicaltrials.gov/.
